# Radiofrequency Ablation and Pulsed Radiofrequency of Suprascapular Nerves for Managing Chronic Shoulder Pain

**DOI:** 10.3390/brainsci15090915

**Published:** 2025-08-26

**Authors:** Alaa Abd-Elsayed, Tristan R. Argall, Lukas J. Henjum, Dustin M. McKindsey, Nathan A. Perkins, Kenneth J. Fiala

**Affiliations:** 1 Department of Anesthesiology, University of Wisconsin, Madison, WI 53707, USA; trargall@wisc.edu (T.R.A.); lhenjum@wisc.edu (L.J.H.); mckindsey@wisc.edu (D.M.M.); naperkins@wisc.edu (N.A.P.); 2 Department of Anesthesiology, University of Michigan, Ann Arbor, MI 48109, USA; fkenneth@med.umich.edu

**Keywords:** suprascapular neuralgia, radiofrequency ablation, chronic pain, suprascapular nerve, pulsed radiofrequency, thermal radiofrequency

## Abstract

Background: Chronic pain is a major contributor to a reduced quality of life in the United States, with chronic shoulder pain comprising a sizeable portion of complaints. Multiple techniques are utilized in the treatment of chronic shoulder pain, but many patients report significant pain refractory to these modalities. For these patients, the suprascapular nerve’s radiofrequency ablation (RFA) offers a potential long-lasting solution. Methods: This retrospective analysis used data from UW Health HealthLink records of patients who received suprascapular RFA from June 2017 to May 2024. Data were collected across 31 procedures, covering demographics, RFA technique, procedure efficacy, duration of relief, adverse events, and relevant medical history. The data were analyzed using a paired *t*-test. Results: The average pre-RFA pain score was 6.08/10, and the average post-RFA pain score was 2.95/10. The average percentage improvement was 63.3%, with a mean duration of improvement of 3.12 months. Five procedures yielded no improvement in pain. Conclusions: This study demonstrated that RFA is an effective alternative therapy for chronic shoulder pain refractory to conventional pain management strategies, with a potential for long-term relief. Limitations of this study are due to the inherent challenges of retrospective analyses.

## 1. Introduction

Approximately 50 million individuals in the United States suffer from chronic pain, defined by the International Association for the Study of Pain as pain persisting for more than three months [1,2]. Chronic pain is associated with a myriad of deleterious health outcomes, including an increased risk of depression, lower satisfaction with life, and reduced scores across quality-of-life metrics, including physical and emotional health [3,4,5]. In addition, chronic pain is particularly burdensome upon the healthcare system. It is one of the most common reasons for emergency department visits and outpatient consultations, which often require extensive workup and repeated interventions [6]. Furthermore, the financial burden that chronic pain imposes upon the United States economy is immense, amounting to approximately $600 billion annually [7].

Chronic shoulder pain is a widespread issue imposing a substantial burden both on healthcare systems and individuals. The lifetime prevalence of this debilitating condition is 66.7%. In comparison, the point prevalence is as high as 26%, with these values increasing in patients who work in more demanding occupations such as construction or other manual labor jobs [8,9]. Similarly, chronic shoulder pain is a major contributor to lost productivity and disability claims, particularly among individuals whose employment involves manual labor or repetitive overhead activities [10].

Suprascapular neuralgia, an often-overlooked cause of chronic shoulder pain, is a neuropathic condition arising from suprascapular nerve irritation [11]. Due to suprascapular neuralgia having overlapping clinical features with more common shoulder disorders such as labral injury, cervical radiculopathy, and the many rotator cuff pathologies, suprascapular neuralgia, especially in its early stages, is commonly unrecognized. When overlooked and untreated, suprascapular neuralgia can lead to progressive nerve dysfunction, refractory pain, muscle atrophy, and progressive nerve dysfunction [12]. In high-risk groups, such as people who perform jobs that include manual labor, but also athletes who perform overhead motions, such as volleyball and baseball players, suprascapular neuralgia has been identified as a major contributor to missed workdays, reduced athletic performance, and chronic shoulder dysfunction [13]. Although the exact prevalence of suprascapular neuralgia remains unclear due to divergences in diagnostic criteria, the existing literature suggests that up to 4% of patients with shoulder pain who are referred to specialty clinics may have suprascapular nerve involvement [14].

The suprascapular nerve arises from the upper trunk of the brachial plexus from the ventral rami of the fifth and sixth cervical nerve roots and is a primary source of shoulder pain [13,15]. After exiting the brachial plexus, it courses through the suprascapular notch beneath the transverse scapular ligament, a common source of its entrapment, and where it is susceptible to traction injuries [13]. Due to its extensive involvement in the musculature and sensory component of key anatomical structures of the shoulder, injury to the suprascapular nerve can cause debilitating sensory and motor dysfunction of the shoulder [13]. The motor component of the suprascapular nerve primarily involves innervation of the supraspinatus and infraspinatus musculature [15]. Its sensory fibers primarily innervate the glenohumeral joint capsule, acromioclavicular joint, and coracohumeral ligament of the shoulder [15]. Additionally, its sensory fibers most commonly branch more proximally along the suprascapular notch, while the motor fibers extend more distally to the suprascapular notch [16]. Insight into the anatomy of the suprascapular nerve is crucial, enabling precise and judicious targeting of its sensory branches while preserving motor function to the infraspinatus and supraspinatus muscles.

Conservative treatment strategies for chronic shoulder pain may include pharmacological intervention, physical therapy, and corticosteroid injections. Additionally, more invasive surgical procedures, such as arthroscopy and rotator cuff repair, are common [17]. In many cases, these therapies may prove effective; however, each may carry suboptimal limitations, especially in patients with neuropathic pain such as suprascapular neuralgia. Pharmacological management with medications such as NSAIDs and anticonvulsants may be poorly tolerated or insufficient to control the patient’s pain. Each carries its side effects as well, including NSAIDs worsening renal function, and anticonvulsants, such as Gabapentin, known to cause dizziness, drowsiness, and confusion among other side effects. Opioid medications have additionally been used, but are also associated with well-known risks, including, and most notably, dependence [18]. Physical therapy can be efficacious in the treatment of musculoskeletal dysfunction through improvement in joint mechanics and strength but often fails to ameliorate pain of neuropathic origin [19]. Corticosteroid injections may also be beneficial; however, repeat injections may weaken tendons and articular cartilage [20]. Surgical approaches to chronic shoulder pain typically entail higher costs, longer recovery times, and potential perioperative complications.

Additionally, surgery has been shown to have poor outcomes in patients without clear structural pathology [21]. The need for alternative modalities is critical for patients with ineffective or contraindicated options. A treatment modality is needed to address the neuralgia aspect of these shoulder injuries, and radiofrequency ablation serves this purpose.

Radiofrequency ablation (RFA) is a minimally invasive procedure to alleviate chronic pain refractory to other modalities. RFA uses thermal energy to induce neurolysis of the nerves in the nociceptive pathways [22]. This technique involves targeted heat application to the nerve via a radiofrequency-emitting electrode, which disrupts afferent pain signaling by destroying nerve fibers, particularly the unmyelinated C fibers and thinly myelinated A-delta fibers [22]. Advances in image-guided procedures have enhanced the precision of RFA, enabling more accurate localization of the target nerve fibers [23]. In this procedure, the ultrasound-guided localization of the suprascapular nerve in the suprascapular notch allows for the selective destruction of sensory nerve fibers. This provides long-lasting analgesia without the systemic effects associated with pharmacologic therapies. There are several methods in which radiofrequency can be used to induce neurolysis. In this study, we examine the conventional form of RFA and a pulsed form of RFA. The traditional form has more reliable neurolysis, but the pulsed form is associated with fewer adverse events [24]. Radiofrequency ablation has already demonstrated benefits in treating neuralgia of several nerves, such as the lumbar, genicular, and cervical nerves [25,26,27].

In addition to its clinical efficacy, RFA offers potential cost advantages. Compared to surgical procedures, which often require general anesthesia, postoperative care, and increased staffing, RFA is typically performed in an outpatient setting at a lower cost [28]. It also avoids the indirect costs of extended rehabilitation, lost work time, and surgical complications. When effective, RFA may reduce the need for repeated corticosteroid injections, long-term pharmacotherapy, or surgical interventions, improving cost-effectiveness and patient quality of life.

RFA has emerged as an effective, minimally invasive treatment option for reducing chronic pain in the suprascapular region [29]. Furthermore, RFA’s low cost, minimal complications, and versatile applications make it a valuable tool for patients suffering from chronic pain [30]. Despite its promise, the current literature on RFA for suprascapular neuralgia remains limited. There is a clear need for additional data to guide the use of RFA in this population, particularly in patients who have failed standard treatments. This retrospective analysis assesses the effectiveness of RFA of the suprascapular nerve in ameliorating chronic shoulder pain resistant to standard treatment modalities. We hypothesize that patients undergoing RFA for chronic shoulder pain secondary to suprascapular neuralgia will experience a significant reduction in pain scores.

## 2. Materials and Methods

### 2.1. Consent and IRB

The University of Wisconsin Institutional Review Board reviewed and exempted this study from full review. Since the study was retrospective and used UW Health Electronic Medical Records (EMRs), no patients were contacted during the study’s development.

### 2.2. Diagnostic Block

All patients in this study were previously diagnosed with chronic shoulder pain that was refractory to conservative treatment measures. Before RFA, each patient was required to have diagnostic and confirmatory blocks of the suprascapular nerve. This procedure was performed under ultrasound guidance. The skin was cleaned using chlorhexidine, sterilized, and anesthetized using a 1% lidocaine solution. A 22-gauge, 3.5″ needle was then inserted. Correct needle placement in the suprascapular notch was confirmed via ultrasound. After negative aspiration, 1% lidocaine or 0.25% bupivacaine was injected. The same technique was used to perform a confirmatory block if the diagnostic block provided more than 50% improvement in pain. Only patients who reported a reduction in pain of greater than 50% following the confirmatory block were deemed appropriate candidates to proceed with RFA, to ensure that the suprascapular nerve was the primary generator of pain symptoms. Individualized modifications to the standard procedural protocol were made on a case-by-case basis at the treating physician’s discretion.

### 2.3. Patient Selection

This study is a retrospective analysis of patients who underwent thermal RFA of the suprascapular nerve for chronic pain from June 2017 to May 2024. Patient data was collected from the UW Health Electronic Medical Records (EMRs) and was analyzed using Microsoft Excel software. Pain scores were recorded in a Visual Analog Scale (VAS), a widely accepted and validated tool for measuring acute and chronic pain [31].

The collected data included patient demographics (age, sex, and BMI), diagnoses, pre-RFA pain score, post-RFA pain score, percentage of pain improvement, date of procedure, duration of improvement, needle size, mode of lesion, adverse events, and relevant medical history. Pain improvement scores were calculated by subtracting the preoperative and postoperative pain scores. Significance was evaluated using a paired *t*-test with an alpha level of 0.05.

For charts that included preoperative and postoperative VAS pain scores but did not report a percentage improvement, the percentage improvement was calculated using the pain scores. For example, a preoperative VAS pain score of 8/10 and a postoperative VAS pain score of 2/10 indicate a 75% reduction in pain. When improvement percentages were recorded without a postoperative VAS pain score, the postoperative pain score was calculated using the reported percentage improvement (i.e., the preoperative pain score was 8/10, and a 25% improvement would indicate a postoperative pain score of 6/10). When a discrepancy existed between the pain scores and the reported percentage improvement, the percentage improvement was preferred, and postoperative pain scores were calculated based on this percentage. For instance, if the pre- and postoperative pain scores were 8/10 and 6/10, respectively, but a 75% improvement was reported, then a postoperative pain score 2/10 was included in the analysis. This approach was chosen because it provides a more accurate representation of the patient’s perceived benefit of the treatment. If a range of pain scores was given, the mean was calculated and used for analysis.

Percentage improvement was calculated only for patients who reported decreased pain scores. It was calculated by subtracting the mean postoperative pain score from the preoperative pain score and dividing the result by the preoperative pain score. The mean duration of pain relief was calculated using procedures that reported an improvement in pain scores. If the duration of improvement was not clearly stated in the medical record, it was calculated as the period from the day the procedure was conducted to the day significant symptoms returned.

Patients who lacked documentation of either preoperative or postoperative pain scores were excluded from the study. Patients were also excluded if they had other procedures performed at or near the RFA site between the recording of pain scores.

### 2.4. RFA Procedure

The suprascapular nerve was identified under ultrasound guidance at the suprascapular notch. The skin was anesthetized with 1% lidocaine. An 18-gauge, 10 mm radiofrequency lesioning needle was inserted under ultrasound guidance until it was on the suprascapular nerve. Sensory and motor testing was performed, confirming tingling in the area of pain, the nerve’s distribution, and a lack of motor activity. Then, after negative aspiration, 2% lidocaine was injected, and RFA was performed. The mode of lesion varied across procedures: 19 procedures utilized a combination of pulsed radiofrequency ablation (PRF) and conventional (lesion mode) ablation, 2 used PRF only (120 s at 42 °C in pulse mode), 2 used conventional (90 s at 42 °C in lesion mode), and in 2 cases, the mode was not documented. Alterations to the standard procedure were made on a patient-by-patient basis.

## 3. Results

Thirty-one suprascapular RFA procedures were reviewed for inclusion in this study. Of these, six procedures were excluded due to incomplete documentation, specifically the absence of pre- or postoperative VAS pain scores or data on percentage improvement. The final analysis included 25 procedures performed across a cohort of 14 unique patients. Several patients underwent more than one procedure during the study period, either due to bilateral shoulder involvement or recurrent symptoms necessitating repeat RFA. These procedures were counted individually in the analysis. Among the 14 patients were 7 males and 7 females, with a mean age of 63.86 ± 19.57 years and a mean BMI of 31.28 ± 7.34 (Table 1).

**Figure 1:**
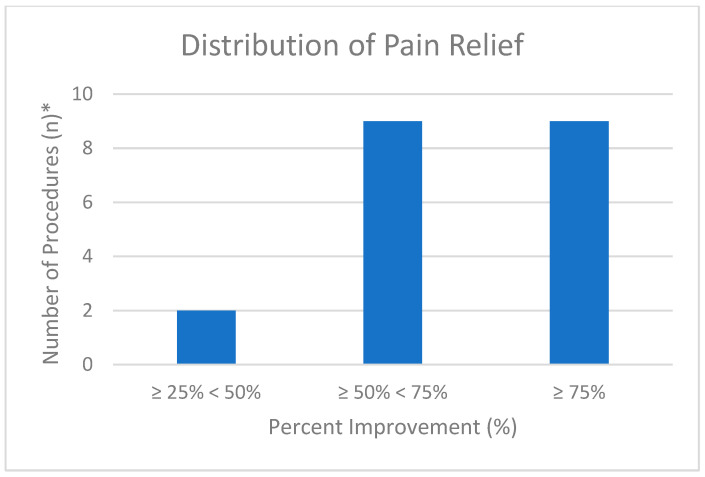
Distribution of pain relief following suprascapular RFA. The number of procedures (*y*-axis) is categorized by the percentage of improvement in pain relief (*x*-axis). * Procedures included only those with a patient-reported improvement greater than 0.0% (*n* = 20).

**Figure 2:**
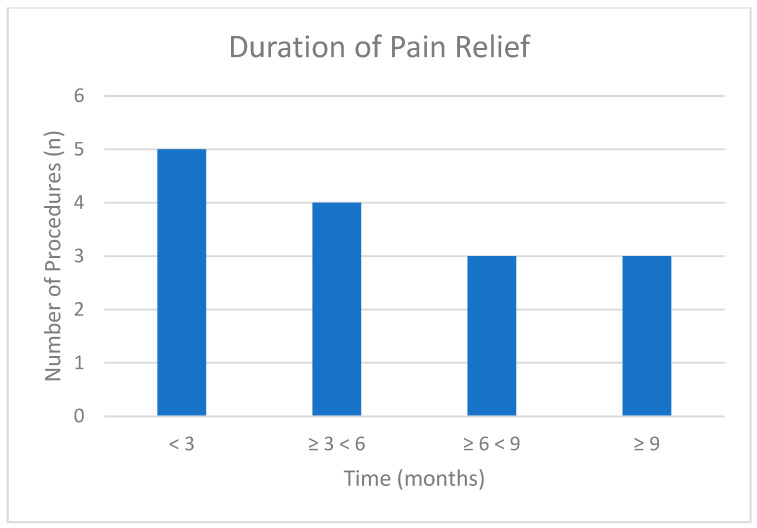
Duration of pain relief following suprascapular RFA. The *x*-axis represents the duration of pain relief categorized into four-time intervals, while the *y*-axis represents the number of procedures yielding each duration of improvement (total *n* = 15).

The mean pre-RFA pain score was 6.08 ± 2.15, and the mean post-RFA pain score was 2.95 ± 2.27 (*n* = 25). A two-tailed paired *t*-test revealed a statistically significant difference in pre- and postoperative pain scores with a *p* < 0.001 (Table 2). Five procedures did not result in any improvement in the patients’ reported pain scores. The mean percentage improvement in the procedures that yielded pain relief (*n* = 20) was 63.3 ± 18.4%. No statistically significant difference was observed between male and female patients (*p* = 0.22). Two procedures resulted in pain improvement of ≥25% and <50%, nine procedures resulted in pain improvement of ≥50% and <75%, and nine procedures resulted in pain improvement of ≥75% (Figure 1). Five procedures resulted in pain relief lasting less than three months, four procedures provided relief for ≥3 and <6 months, three procedures resulted in pain relief lasting from ≥6 to <9 months, and three procedures demonstrated relief lasting ≥9 months (Figure 2).

Out of the total 31 procedures reviewed in this study, two adverse events were recorded (6.5%). Both were temporary postoperative pain flares that were resolved spontaneously over time without the need for further medical intervention. No significant, long-lasting adverse events were recorded.

## 4. Discussion

The findings of this study provide robust evidence for the efficacy of RFA in treating suprascapular neuralgia, reinforcing its utility as a viable treatment for chronic shoulder pain. Statistical analysis demonstrated a significant reduction in pain scores following RFA (Table 2, *p* < 0.001), which supports our hypothesis that suprascapular RFA effectively reduces pain. The impact of RFA is further demonstrated by an average pain reduction of more than 50% (Table 2), with nine procedures achieving a pain improvement of 75% or greater (Figure 1) among the 20 procedures that met the inclusion criteria for the improvement calculation. Furthermore, these data demonstrate the long-lasting effect of suprascapular RFA, as 10 out of 15 patients had pain relief for ≥3 months (Figure 2). Overall, these outcomes provide strong evidence that RFA of the suprascapular nerve is an effective therapeutic option for patients experiencing chronic shoulder pain due to suprascapular neuralgia.

This data contributes to the existing literature on radiofrequency ablation for treating suprascapular neuralgia. Luleci et al. reported a significant reduction in pain intensity in 74% of patients after treatment [32]. This finding is consistent with our results and those of several other studies, including a systematic review that identified 29 studies initially [33]. While variability exists regarding the degree and duration of pain relief, particularly among retrospective studies, a consistent trend toward benefit is observed. In a retrospective analysis by Gabrhelik et al. evaluating the effect of PRF, patients reported at least a 50% reduction in pain [34]. Our findings are similar, with a 63.3% reduction in pain in our cohort. The use of combined pulsed and conventional RFA in our study was made using clinical judgment by the providing physicians, leveraging the benefits of each method. Conventional RFA has been shown to provide longer-lasting pain relief, while PRF reduces adverse events [24]. Although in total, we report only two adverse events out of thirty-one procedures, highlighting the safety of this procedure. Direct evidence for the combined RFA approach regarding the suprascapular nerve is limited; a meta-analysis on trigeminal neuralgia by Orhurhu et al. found that combined RFA of the trigeminal nerve provided better long-term pain relief and fewer adverse outcomes than either modality alone [35]. Discrepancies in pain relief across studies could be due to variations in RFA techniques, procedural settings, or sample sizes, but they underscore the general efficacy of the approach. Further research is required to elucidate the role of different RF lesioning techniques in the treatment algorithm for chronic shoulder pain caused by suprascapular neuralgia.

Our patient selection criteria, which required successful diagnostic and confirmatory nerve blocks, likely increased the precision of nerve targeting and contributed to the overall treatment response. Additionally, ultrasound guidance enables real-time visualization of the nerve, enhancing precision, reducing procedure variability, and lowering the radiation levels to which patients are exposed compared to other imaging methods, such as fluoroscopy [36]. Targeting the sensory branches of the suprascapular nerves with fidelity is paramount to limiting motor deficits and enhancing functional outcomes. Avoiding the motor branches of the suprascapular nerve is crucial for preserving rotator cuff function and maintaining shoulder mechanics after the procedure. This is of particular importance for patients who require upper limb strength, such as those whose jobs involve manual labor and athletes, who are also patients commonly afflicted with suprascapular neuralgia [13].

Suprascapular neuralgia is one of many conditions responsible for chronic shoulder pain. Oftentimes, chronic shoulder pain is multifactorial, with cervical radiculopathy, myofascial pain, and glenohumeral arthropathy among other conditions acting in conjunction with suprascapular neuralgia [12]. Therefore, if necessary, a comprehensive diagnostic approach, including diagnostic blocks and a thorough physical exam and imaging, is essential to isolate the pain generator and target therapies effectively. Furthermore, as with chronic pain, more broadly, psychosocial factors, including depression and anxiety, to name a few, can profoundly influence pain perception and treatment outcomes [37]. In our study, these influences were not formally assessed; however, future prospective studies should examine their impact on outcomes and incorporate validated psychometric tools to quantify psychological or behavioral factors that may modulate treatment response.

The two postoperative pain flares observed in our cohort are consistent with adverse events noted in prior studies. For instance, in Eckmann et al., temporary postoperative discomfort was noted, with a very low incidence of serious complications [38]. Risk is expected in all invasive procedures; however, with minimally invasive procedures such as RFA, those risks are typically self-limiting and often resolve spontaneously over time, as in our study, with both patients’ symptoms remitting within two weeks post-procedure. Our findings contribute to the expanding evidence that RFA is an effective, minimally invasive treatment for suprascapular neuralgia. However, additional research is necessary to more precisely evaluate which patient demographics are most susceptible to adverse events and thus minimize them.

The variation in duration of pain relief observed across our cohort is consistent with known mechanisms of nerve regeneration and central sensitization observed after radiofrequency ablation. Nerve fibers may regenerate over time, particularly if incomplete lesioning or adjacent nerves contribute to overlapping sensory territories [39]. Additionally, patients with longer durations of pre-treatment pain may develop neuroplastic changes that diminish the efficacy of peripherally targeted therapies. Identifying clinical or imaging markers that predict long-term response could help optimize patient selection and improve overall treatment outcomes.

The primary limitations of this study arise from the challenges inherent in retrospective analysis. A lack of follow-up and inconsistent documentation can affect data quality. The lack of patient follow-up may be due to several factors, including complete resolution of pain, no improvement in pain, relocation, changes in healthcare systems, and changes in insurance. Additionally, our study spanned the years 2017–2024, and therefore, we infer that some patients were lost to follow-up due to COVID-19 restrictions [40]. To minimize bias in patient-reported outcomes, we ensured that physicians performing the RFA were not the same as the provider collecting post-procedural outcomes.

Patients often experience suprascapular neuralgia in concert with other musculoskeletal or neurologic pathology in the shoulder, which can affect the response to treatment. Additionally, patients with chronic medical conditions could have differing responses to RFA treatment. To address this, we limited our analysis to patients whose shoulder pain was refractory to other treatment modalities and was confirmed to be suprascapular neuralgia via diagnostic block. While other pathologies may be present, our analysis examined the efficacy of RFA in treating the suprascapular neuralgia. Further research should be conducted to evaluate the effectiveness of RFA in treating suprascapular neuralgia in the context of various pathologies.

Patients often undergo multiple therapies concurrently and use various pain medications, complicating the attribution of outcomes to a single treatment. However, RFA is only indicated in cases where pain is refractory to other modalities. These patients have already failed more conservative measures and were diagnosed via confirmatory nerve block with suprascapular neuralgia, increasing the likelihood that changes in pain scores are attributable to RFA as opposed to other treatments. To further overcome these challenges, a systematic approach to data collection was employed, involving students who were not involved in the clinic settings where procedures occurred to collect data. Additionally, all our patients received their healthcare at one institution, ensuring consistent and accurate documentation.

Another limitation of this study is the relatively small size of our cohort, which restricts the generalizability of our findings and the statistical power of our analysis. This also limits the ability to conduct subgroup analyses, such as comparisons of RFA modality or confounding pathology. Further research should aim to conduct prospective randomized controlled trials with standardized protocols, stratified patient cohorts, and more cases to compare the therapeutic effects of pulsed and conventional radiofrequency ablation of the suprascapular nerve for patients with chronic shoulder pain.

The incorporation of patient-reported outcome measures focusing on function and disability in addition to pain could prove beneficial to incorporate in future studies, to further characterize the benefit of the procedure on patient outcomes. To this end, the Shoulder Pain and Disability Index (SPADI) and quality-of-life metrics could provide a more comprehensive assessment than pain scores alone [41]. Additionally, cost-effectiveness analyses comparing RFA to repeat corticosteroid injections, surgical interventions, and pharmacological therapies would be valuable for informing clinical decision-making and insurance reimbursement policies. Given that nerves targeted during the procedure may regenerate over time, assessing the effectiveness of repeat RFA treatments in patients with recurrent pain is essential. Further research is warranted to fully understand the mechanisms and pathways involved in RFA, including more detail on the mechanistic differences between the various RFA methods.

## 5. Conclusions

Our findings support the efficacy of RFA as a minimally invasive treatment for chronic shoulder pain secondary to suprascapular neuralgia that is refractory to more conservative therapies. In juxtaposition to other pain management modalities, our findings suggest that RFA offers a substantial and durable analgesic benefit with minimal procedural risk, making it an attractive option for patients who have exhausted other treatment methods.

## Figures and Tables

**Table 1:** Demographics.

Total RFA (*n*)	25
Total patients (*n*)	14
Sex (M:F)	7:7
Age (years)	63.86 ± 19.57
BMI (kg/m^2^)	31.28 ± 7.34

BMI body mass index.

**Table 2:** Summary of results.

VAS pain score pre-RFA (*n* = 25)	6.08 ± 2.15
VAS pain score post-RFA (*n* = 25)	2.95 ± 2.27
% improvement (*n* = 20) *	63.3 ± 18.4
% improvement by sex *	Male: 58.6 ± 16.9 (*n* = 11) Female: 69.1 ± 19.5 (*n* = 9) *p* = 0.22

Data are presented as mean ± SD, with the total number represented by the *n* value. VAS refers to the Visual Analog Scale. * Procedures included only those with a patient-reported percentage improvement greater than 0.0%.

## Data Availability

No new data were created or analyzed in this study. Data sharing is not applicable to this article.

## Abbreviations

The following abbreviations are used in this manuscript:

RFA	Radiofrequency Ablation
NSAID	Non-Steroidal Anti-Inflammatory Drug
PRF	Pulsed Radiofrequency Ablation
VAS	Visual Analog Score
